# Color differences among feral pigeons (*Columba livia*) are not attributable to sequence variation in the coding region of the melanocortin-1 receptor gene (MC1R)

**DOI:** 10.1186/1756-0500-6-310

**Published:** 2013-08-05

**Authors:** Romain Derelle, Fyodor A Kondrashov, Vladimir Y Arkhipov, Hélène Corbel, Adrien Frantz, Julien Gasparini, Lisa Jacquin, Gwenaël Jacob, Sophie Thibault, Emmanuelle Baudry

**Affiliations:** 1 Centre for Genomic Regulation (CRG), Dr. Aiguader 88, Barcelona 08003, Spain; 2 Universitat Pompeu Fabra (UPF), Barcelona 08003, Spain; 3 Institució Catalana de Recerca i Estudis Avançats (ICREA), Pg. Lluís Companys 23, Barcelona 08010, Spain; 4 Institute of Theoretical & Experimental Biophysics, Russian Academy of Sciences, Pushchino, Moscow Region, Russia; 5 Laboratoire Ecologie & Evolution (EcoEvo), UMR 7625, CNRS UPMC ENS, Université Pierre et Marie Curie (UPMC), Paris, France; 6 Department of Biology, McGill University, Montréal, Québec, Canada; 7 Laboratoire Ecologie, Systématique et Evolution, CNRS-UMR 8079, Université Paris-Sud, Orsay Cedex F-91405, France

**Keywords:** *Columba livia*, Feral pigeon, MC1R, Pigmentation, Color, Birds

## Abstract

**Background:**

Genetic variation at the melanocortin-1 receptor (MC1R) gene is correlated with melanin color variation in many birds. Feral pigeons (*Columba livia*) show two major melanin-based colorations: a red coloration due to pheomelanic pigment and a black coloration due to eumelanic pigment. Furthermore, within each color type, feral pigeons display continuous variation in the amount of melanin pigment present in the feathers, with individuals varying from pure white to a full dark melanic color. Coloration is highly heritable and it has been suggested that it is under natural or sexual selection, or both. Our objective was to investigate whether MC1R allelic variants are associated with plumage color in feral pigeons.

**Findings:**

We sequenced 888 bp of the coding sequence of MC1R among pigeons varying both in the type, eumelanin or pheomelanin, and the amount of melanin in their feathers. We detected 10 non-synonymous substitutions and 2 synonymous substitution but none of them were associated with a plumage type. It remains possible that non-synonymous substitutions that influence coloration are present in the short MC1R fragment that we did not sequence but this seems unlikely because we analyzed the entire functionally important region of the gene.

**Conclusions:**

Our results show that color differences among feral pigeons are probably not attributable to amino acid variation at the MC1R locus. Therefore, variation in regulatory regions of MC1R or variation in other genes may be responsible for the color polymorphism of feral pigeons.

## Findings

Plumage color variation in birds, within or between species, has long attracted the attention of evolutionary biologists and ecologists because of its potential importance in crypsis, adaptation in general, sexual selection and speciation [1]. Within species, plumage color variation is also an interesting example to study selective mechanisms for maintaining polymorphism in natural populations [2,3]. Feral pigeons show extreme plumage variation, which originated from artificial selection of domestic stock [4]. They show two major types of melanin-based colorations: black coloration, due to the deposition of eumelanic pigments in the feathers, and a less frequent ash-red coloration, due to the deposition of pheomelanic pigments [5]. Within each color type, feral pigeons also display continuous variation in the amount of melanin pigment present in feathers [4], from pure white to a full dark melanic coloration. Coloration in pigeons is highly heritable [4,6] and is only weakly influenced by environmental conditions [4]. Crosses have shown that melanin type variation is controlled by one locus on the sex chromosome, whereas the amount of melanin pigment in feathers is mainly controlled by one autosomal locus called “C locus” with numerous alleles [4].

The melanin-based colors of pigeons covary with several life-history traits, suggesting that differently colored individuals may have different fitness in alternative environments, which could explain the maintenance of polymorphism in this species [2]. For example, when facing high or low food availability, differently colored feral pigeons show different responses regarding body mass maintenance, egg laying, and offspring quality [7]. It has been also shown that immune responsiveness and parasite intensity correlates with coloration in feral pigeons [8]. Furthermore, mate choice is strongly influenced by plumage coloration and pattern, indicating that the trait is probably also under sexual selection [4]. To test whether plumage colors of pigeons has been shaped by natural selection, it would be interesting to identify genes controlling colors in pigeons, to detect possible molecular footprints of selection among those genes.

The melanocortin 1 receptor gene (MC1R) encodes a seven-transmembrane domain G-protein-coupled receptor expressed primarily in melanocytes of developing feathers and hair [1]. MC1R can influence the type and amount of melanin produced in developing feathers [9]. Multiple studies have linked amino acid variability of MC1R to different plumage color in wild bird populations. MC1R variation is for example correlated with the plumage polymorphism of the bananaquit, *Coereba flaveola*, and the snow goose, *Anser c. caerulescens*[10], the arctic skua, *Stercorarius parasiticus*[11], the red-footed booby, *Sula sula*[12], the gyrfalcon, *Falco rusticolus*[13,14] and Eleonora’s falcon, *Falco eleonorae*[15]. The aim of our study was to determine whether MC1R variation is also correlated with plumage polymorphism in feral pigeons. In particular, we hypothesized that MC1R may be the autosomal “C locus” that control the amount of melanin produced in pigeons.

**Figure 1:**
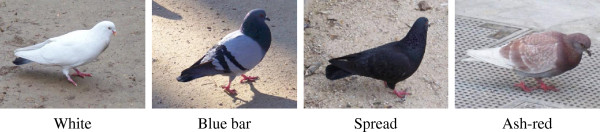
**Four plumage categories of the studied feral pigeons: white or almost white (pigeons that have a pure white color over almost all the body, but show a few feathers of a different color), blue bar (wild type), spread (melanic) and ash-red.** The three first categories correspond to an increasing production of eumelanin, whereas the last one corresponds to a production of pheomelanin.

We chose individuals with four different types of plumage (Figure 1): white or almost white (i.e. white over almost all the body but with a few feathers of a different color), blue bar (wild type: gray mantle with two dark wing bars), spread (a completely melanic plumage) and ash-red. The three first categories correspond to an increasing production of eumelanin, whereas the last one corresponds to a production of pheomelanin. Blood samples were collected from free-living pigeons in Paris, France and Saratov, Russia (Table 1). Genomic DNA was extracted from the blood samples with a Macherey Nagel Nucleospin tissue kit, following the manufacturer’s protocol. Extracted DNA was resuspended in 100 μl elution buffer.

**Table 1:** Plumage, geographic origin and MC1R sequence of the studied feral pigeons

**Individual bird identification**	**Color**	**Geographic origin**^**a**^	**MC1R polymorphic sites and their positions**^**b, c**^
			*** *******
			111112233588
			012494524024
			356192333993
Consensus			GCCGCCGCGGTT
G8	White	P	........A...
G6		P	............
JJ		P	............
B04	Blue bar	P	............
B15		P	............
J18		P	............
JF17		P	......R.....
S9	(wild type)	S	A..A.G.A....
p1		S	............
L167		S	.TG.........
J17	Spread	P	............
V15		P	............
V24		P	......R.....
O18		P	........R...
S3		S	............
S4		S	..G.A.......
S5	(melanic)	S	............
S6		S	............
S7		S	..........A.
l20		S	...........C
l30		S	......A..A..
l35		S	......A.....
V4	Ash-red	P	............
V5		P	............
V17		P	............

^a^: geographic origin indicated as “P” for Paris, France, and “S” for Saratov, Russia.

^b^: the chicken MC1R sequence was used as a reference for the position of the polymorphic nucleotides.

^c^: the IUPAC ambiguity code was used to indicate cases where the sequenced individuals were heterozygous.

* indicates a non-synonymous change.

A segment of the MC1R coding sequence was amplified using the conserved primers MSHR72 [11] and MSHR78 [16]. Each PCR reaction was run in a 20 μl volume containing 1 μl of DNA solution, 400 μM of each dNTP, 1.75 μM of Mg^++^, 1 μM of each primer and 1.25 units of Taq polymerase (Qiagen). Thermocycle conditions were 94°C for 30 s, 58°C for 30 s, and 72°C for 1 min, for a total of 35 cycles. Purified template DNA was sequenced on both strands using standard sequencing techniques, with the PCR primers and with internal primers designed for *C. livia*: MC1R-144 F (5’-TGGTGAGCCTGGTGGAGAAC-3’) and MC1R-773R (5’-CAGGTGACGATGAGGATGAG-3’). All sequences were proof-read and aligned manually. Heterozygotes were identified by visual inspection of chromatograms and confirmed by sequence from both DNA strands. Sequences were deposited in the GenBank database [GenBank: KC819623- KC819647].

We obtained a final alignment of 888 bp, corresponding to positions 50 to 940 of the 945 bp chicken MC1R coding sequence (Table 1). We observed a three base pair deletion, corresponding to a one amino acid deletion at amino acid position 226 of the chicken MC1R protein. This deletion is observed among all studied *C. livia* and is therefore not related to the color polymorphism of the feral pigeons. Among 25 individuals, we detected 15 non-synonymous substitutions at 10 sites in 10 individuals and two synonymous substitutions at 2 sites in 2 individuals, but none of them were associated with a plumage type. Surprisingly, one of these non-synonymous substitutions which appeared in 4 individuals, the Val85Met point substitution (nucleotide position 253), has been previously shown to be associated with melanic phenotypes in the lesser snow goose (*Anser c. caerulescens*) [10] and in the red-footed booby (*Sula sula*) [12], but no such association is observed in our feral pigeon sample.

We did not sequence the first 17 codons of MC1R and, therefore, cannot exclude the possibility that variants linked to the coloration of feral pigeons were present among these codons. However, to our knowledge, no study has reported a relationship between melanic variation and sequence variation in this initial part of the coding region of MC1R (see for example Mundy *(*2005) for a review of the position of the MC1R sequence variants associated with plumage and hair colour change in birds and mammals). Our results therefore strongly suggest that color differences among feral pigeons are not attributable to amino acid variation at the MC1R locus in the two populations where our samples come from. Variations in regulatory regions of MC1R or in other genes are probably responsible for the color polymorphism of these feral pigeons. Within a species, the genetic determinism of color variation might differ between populations (e.g. [17]) and our result might therefore not be generalizable to all feral pigeon populations. Note however that our results are in agreement with a recent study by Miller and Shapiro [18], which showed that MC1R genotypes are not associated with plumage color variation in the domestic rock pigeon.

Multiple studies have linked melanic coloration variation in birds to changes in the coding sequence of MC1R (see for example [13] for a recent list). Other studies, for example [19-21] and this study on feral pigeons did not find such an association. This is to be expected given that over 150 genes that affect animal color and patterning have been identified [22]. Until recently, due to the relatively high cost of sequence data in non model species, studies on the genetic architecture of color polymorphism were usually performed by analyzing the variability at one locus, usually MC1R, or a few candidate genes known to be frequently involved in pigmentation variation. The development of next-generation sequencing technologies, by increasing the speed and throughput capacities of DNA sequencing, has dramatically reduced overall sequencing costs [23]. In the near future it may become possible to screen whole genomes in non-model species [24] and further elucidate the genetic architecture of color polymorphism in many more species.

## Competing interests

The authors declare that they have no competing interests.

## Authors’ contributions

Conceived and designed the experiments: RD, VYuA, FAK, EB. Performed the experiments: RD, ST. Analyzed the data: RD, EB. Contributed reagents/materials/analysis tools: VYuA, FAK, HC, AF, JG, LJ. Wrote the paper: EB. All authors read and approved the final manuscript.
